# To: Closing the critical care knowledge gap: the importance of publications from low-income and middle-income countries

**DOI:** 10.62675/2965-2774.20250026

**Published:** 2025-05-13

**Authors:** Vanessa Soares Lanziotti, Lazaro Nelson Sanchez-Pinto

**Affiliations:** 1 Universidade Federal do Rio de Janeiro Instituto de Puericultura e Pediatria Martagão Gesteira Clinical Research Unit and Pediatric Intensive Care Unit Rio de Janeiro RJ Brazil Clinical Research Unit and Pediatric Intensive Care Unit, Instituto de Puericultura e Pediatria Martagão Gesteira, Universidade Federal do Rio de Janeiro - Rio de Janeiro (RJ), Brazil.; 2 Ann & Robert H. Lurie Children's Hospital of Chicago Chicago Illinois United States Ann & Robert H. Lurie Children's Hospital of Chicago - Chicago, Illinois, United States.; 3 Northwestern University Feinberg School of Medicine Departments of Pediatrics and Preventive Medicine Chicago Illinois United States Departments of Pediatrics and Preventive Medicine, Northwestern University Feinberg School of Medicine - Chicago, Illinois, United States.

To the Editor

We read with great interest the recent editorial published by Critical Care Science, "Closing the critical care knowledge gap: the importance of publications from low-income and middle-income countries",^(1)^ a necessary and timely article about the importance of critical care research from low and middle-income countries (LMIC) and the existent barriers to conducting and publishing such research. Different realities often lead to different healthcare priorities, policies, and interventions, yet the LMIC perspective is often missing from critical care literature.

Pediatric sepsis is a good example of this problem. Although most of sepsis deaths in children occur in LMICs - the chance of a child dying from sepsis being four times higher than in high-income countries (HICs) - the vast majority of research comes from HICs.^(2)^ Sepsis presents particular diagnostic and treatment challenges in LMICs due to resource limitations, higher disease burden, and differing etiologic agents compared to HICs. Considering this context, many products of the HIC literature, such as epidemiological studies, care guidelines, and screening criteria, are not applicable in LMICs. In the case of pediatric sepsis, this need for increased contextualization has been widely discussed in recent years,^(3)^ and this has resulted in some promising advances. These include developing and publishing a consensus guideline for managing pediatric sepsis specifically focused on the Latin American region.^(4)^ Likewise, knowing the epidemiological reality of pediatric sepsis in countries in the global south is essential, and as the editorial accurately described "collective efforts surpass individual contributions".^(1)^ In Brazil, the Sepsis PREvalence Assessment Database in Pediatric population (SPREAD PED) is an excellent example of a collaborative national epidemiological study^(5)^ and was the inspiration for a similar epidemiological study recently conducted in the larger Latin American region, which should be published in the coming months. These collaborative efforts are crucial for closing the knowledge gap in pediatric sepsis and could provide invaluable data on epidemiological, clinical presentation, and outcomes in LMIC settings.

Stronger cross-talk and bidirectional learning between LMIC and HIC researchers and experts is also necessary. In pediatric sepsis, the new Phoenix criteria for pediatric sepsis and septic shock, published in 2024, was a data-informed, international consensus that included experts from around the world, both from HICs and LMICs, including representatives from Latin America, Africa, and Asia.^(6)^ Furthermore, the new Phoenix criteria were derived and validated using data from over 3.6 million pediatric hospital encounters from the United States, Colombia, Kenya, Bangladesh, and China.^(7)^ These criteria were shown to have improved performance in discriminating children with infections at higher risk for poor outcomes in both HICs and LMICs compared to the prior consensus criteria from 2005.^(6)^ This improved generalizability across HICs and LMICs - which was only possible thanks to the extensive international collaboration - provides a new benchmark for equitable research in pediatric sepsis worldwide.

The example of pediatric sepsis, including the development of new Phoenix criteria and the clinical guidelines and epidemiological studies being carried out in Latin America, should serve as inspiration for continuing to close the critical care knowledge gap in other conditions. This is just the beginning of a long road ahead. We thank the authors of the editorial for bringing this topic to light, and we hope this discussion can continue to catalyze advancements in research for critically ill patients worldwide.
